# Hypoxia inducible factor-1 alpha and prolinhydroxlase 2 polymorphisms in patients with severe sepsis: a prospective observational trial

**DOI:** 10.1186/s12871-016-0225-y

**Published:** 2016-08-11

**Authors:** Annika Höcker, Miriam Rabeling, Alexandra Bick, Linda Cox, Maximiliane Kreuzer, Andrea Engler, Kai Walstein, Hagen S. Bachmann, Karl-Heinz Jöckel, Lewin Eisele, Michael Adamzik, Jürgen Peters, Simon T. Schäfer

**Affiliations:** 1 Klinik für Anästhesiologie und Intensivmedizin, Universitätsklinikum Essen and Universität Duisburg-Essen, Hufelandstraße 55, D-45122 Essen, Germany; 2 Institut für Pharmakogenetik, Universitätsklinikum Essen and Universität Duisburg-Essen, Essen, Germany; 3 Institut für Medizinische Informatik, Biometrie und Epidemiologie, Universitätsklinikum Essen and Universität Duisburg-Essen, Essen, Germany

**Keywords:** Sepsis, Genetic variants, Hypoxia inducible factor, HIF, Polymorphism

## Abstract

**Background:**

Hypoxia-inducible-factor-1α (HIF-1α) and HIF-1 degrading prolyl-hydroxylases (PHD) are key regulators of the hypoxic-inflammatory response. Functionally active genetic variants in the *HIF-1α* (C/T; Single Nucleotide Polymorphism (SNP) rs11549465) and the *PHD2* gene (*EGLN1*; C/T; SNP rs516651 and T/C; SNP rs480902) are associated with altered HIF-1α mRNA nuclear translocation and an altered adaptation to hypoxia. Furthermore, the HIF system is important in surviving inflammatory disorders and sepsis. Thus, we tested the hypotheses, that SNPs in the *HIF-1α* or *PHD2* genes are (1) common in Caucasians, with 2) the *HIF-1α* genetic variant being associated with an altered HIF-1α mRNA expression; and 3) independent risk factors for 30-day mortality in severe sepsis.

**Methods:**

After ethics approval, 128 septic patients (Caucasian descent) were included prospectively within 24 h after first diagnosing sepsis. Patients characteristics and severity of illness (simplified acute physiology score II), genotypes (Taqman assay), and their influence on leukocyte HIF-1α-mRNA-expression (Real-Time PCR) and 30-day mortality were determined.

**Results:**

Frequencies were 0.8 % for homozygous *HIF-1α* TT-carriers (CT 17.6 %; CC 81.6 %), 2.5 % for homozygous *PHD2* SNP rs516651 TT-allele carriers (CT 17.5 % and CC 80 %), and 9.4 % for homozygous *PHD2* SNP rs480902 TT-allele carriers (CT 34.4 % and CC 56.3 %). While *HIF-1α* T-allele carriers had a borderline decrease in HIF-1α-mRNA-expression (*p* = 0.06) neither *HIF-1α* nor *PHD2* SNPs were (independent) risk factors for 30-day mortality.

**Conclusions:**

Genetic variants in *HIF-1α* and *PHD2* genes exist in Caucasians but do not appear to alter 30-day mortality in sepsis.

## Background

Hypoxia inducible factor-1α (HIF-1) and HIF-1 degrading prolyl-hydroxylases (PHD) are key regulators of the hypoxic response but are also involved in many cellular actions [1]. Furthermore, we could recently show that leukocyte HIF-1α-mRNA-expression is decreased in severe sepsis and inversely correlated with disease severity [2]. Thus, genetic variants in *HIF-1* or HIF-degrading *PHD2* could impact on HIF-1α mRNA expression and outcome in septic patients.

Recently, functionally active genetic variants in the *HIF-1α* (C/T; Single Nucleotide Polymorphism (SNP) rs11549465) and the *PHD2* gene (*EGLN1*; C/T; SNP rs516651 and T/C; SNP rs480902) were identified [3–5] and were associated with increased HIF-1α nuclear translocation and a worse outcome compared to the TT-genotype in several tumors [6]. However, the HIF-1-pathway plays an important role in adaptation to inflammation, too, as both HIF-1α mRNA and intracellular HIF-1α protein are highly increased following inflammatory stimuli [7, 8]. With persistent inflammation, HIF-1α is suppressed in vitro and even in patients with sepsis, possibly to counter regulating an overwhelming inflammatory response [2, 7, 9]. Furthermore, the *HIF-1α* T-allele is associated with an adverse outcome in acute injury of the kidney, i.e. an organ with a HIF-mediated oxygen sensor, important in mediating erythropoiesis [1, 10]. In high altitude populations, the *PHD2* variant rs480902 CC-genotype is associated with an increased heart rate and higher oxygen saturation under hypoxia, and *PHD2* variant rs516651 T-allele carriers are associated with a decreased incidence of acute mountain sickness [5]. Furthermore, PHD deficiency is associated with an altered inflammatory response, i.e., suppressed cytokine expression in mice [11].

Taken together, *HIF-1α* and *PHD2* genetic variations are functionally active but their impact on critical illness has not yet been investigated. Accordingly, we examined potential effects of genetic variants of *HIF-1* and HIF degrading *PHD2*, in severe sepsis. Specifically, we tested the hypotheses, that SNPs in the *HIF-1α* or *PHD2* genes are 1) common in Caucasians with 2) the *HIF-1α* genetic variant being associated with an altered HIF-1α mRNA expression, and 3) independent risk factors for 30-day mortality in severe sepsis.

## Methods

The study was reviewed and approved by the Medical Faculty’s ethic committee (no. 06-3078, University of Duisburg-Essen, Essen, Germany). Adult patients with sepsis admitted to an intensive care unit of the University Hospital Essen, Germany, between 2010 and 2014 were evaluated for inclusion. Patients were considered eligible when they fulfilled the sepsis criteria as defined by Bone et al. [12]. Patients who refused or withdrew study participation, underage persons, and all individuals with non-Caucasian ethnicity were excluded.

Arterial blood samples were taken for blood tests, microbiology cultures, genotyping, and mRNA extraction within the first 24 h after diagnosing sepsis. Furthermore, SAPS II (Simplified Acute Physiology Score II), length of hospitalization, and 30-day mortality were recorded [2, 13].

The characteristics both of septic patients stratified for either genetic variant are displayed in Table 1 (*HIF-1α*), Table 2 (*PHD2* rs516651), and Table 3 (*PHD2* rs480902), respectively.

**Table 1:** Clinicopathologic characteristics of septic patients with genetic variants in the Hypoxia-inducible factor-1α gene (SNP rs11549465)

	*HIF 1α* CC	*HIF 1α* CT	*HIF 1α* ^a^ TT	*p*-value
	*n* = 102	*n* = 22	*n* = 1	
Patients’ characteristics				
Gender (women/men; %)	37/65 (36.3/63.7)	4/18 (18.2/81.8)	1/0	0.103**
age (years; median; Q1; Q3)^c^	56 (48–66)	68 (53–72)	68	0.027*
height (cm; median; Q1; Q3)^c^	172 (165–180)	177 (167–181)	160	0.395*
body weight (kg; median; Q1; Q3)^c^	80 (68–91)	84 (72–92)	80	0.496*
BMI (kg m-^2^; median; Q1; Q3)^c^	26.2 (22.9–30.8)	27.9 (22.7–29.7)	31.3	0.741*
Heart rate (min^−1^; median, Q1; Q3)^c^	100 (90–125)	107 (85–132)	101	0.870*
Mean arterial blood pressure (mmHg; median; Q1; Q3)^c^	78 (63–90)	70 (63–79)	70	0.200*
creatinin serum concentration (mg dl^−1^; (median; Q1;Q3)^c^	1.43 (1.0–2.39)	1.71 (0.91–2.58)	2.48	0.749*
Dialysis (yes/no; %)	67/34 (65.7/33.3)	16/6 (72.7/27.3)	0/1	0.564**
Primary diagnoses %				
Gastrointestinal disease	25 (29.8)	10 (45.5)	1	^d^
Lung disease	21 (25)	4 (18.2)		
Cardiovascular disease	7 (8.3)	3 (13.6)		
Hematooncological disease	6 (7.1)	1 (4.5)		
Urogenital cancer	6 (7.1)	1 (4.5)		
Intraabdominal pathology, other Cancer, other	19 (22.6)	3 (13.6)		
Infectious variables				
White blood cell count (10^9^ l^−1^; median; Q1; Q3)^c^	14.1 (9.8–20.1)	11.2 (6.4–17.0)	18.8	0.220*
Procalcitonin concentration (μg l^−1^; median; Q1; Q3)^c^	3.0 (1.0–19.2)	3.7 (0.6–9.2)	16.1	0.614*
C-reactive protein concentration (g l^−1^; median; Q1; Q3)^c^	17.2 (8.8–26.3)	18 (9.7–25.0)	5.6	0.627*
Disease severity				
SAPS II^e^ (mean ± SD)^b^	35 (27–48)	40 (33–50)	41	0.248*
Hospital stay (d; mean ± SD)^b^	23 (13–37)	21 (14–39)	67	0.729*
30-day mortality (%)	37 (36.3)	10 (45.5)	0	0.502**

Biometric data, primary diagnoses, infectious variables, and disease severity of 125 septic patients. Data were documented at the time of first diagnosing sepsis

*Numbers; *p*-value based on Mann-Whitney-*U* test

**Numbers; *p*-value based on Pearson-Chi-squared tests

^a^no statistical test has been applied for comparison of TT genotypes with CT or CC genotypes due to sample size of *n* = 1

^b^mean ± standard deviation

^c^median with 25 and 75 % quartiles (median; Q1; Q3);

^d^no statistical test has been applied here

^e^Simplified Acute Physiology Score II

**Table 2:** Clinicopathologic characteristics of 120 septic patients with genetic variants in the prolyl-hydroxlase 2 gene (SNP rs516651)

	*PHD2* CC	*PHD2* CT	*PHD2* TT	*p*-value
	*n* = 96	*n* = 21	*n* = 3	
Patients characteristics				
Gender (women/men; %)	38/58 (39.6/60.4)	5/16 (23.8/76.2)	1/2 (33.3/66.7)	0.094**
age (years; median; Q1; Q3)^b^	55 (44–68)	58 (53–65)	71 (50–71)	0.201*
height (cm; median; Q1; Q3)^b^	172 (165–180)	179 (170–184)	185 (156–185)	0.342*
body weight (kg; median; Q1; Q3)^b^	80 (65–90)	90 (77–110)	78 (75–78)	0.205*
BMI (kg/m^2^; median; Q1; Q3)^b^	26.2 (22.8–30.8)	28.1 (25.3–34)	26.3 (22.8–26.3)	0.434*
Heart rate (min^−1^; median, Q1; Q3)^b^	100 (90–121)	113 (94–156)	90 (70–90)	0.063*
Mean arterial blood pressure (mmHg; median; Q1; Q3)^b^	75 (63–87)	73 (61–87)	76 (63–76)	0.850*
creatinin serum concentration (mg dl^−1^; (median; Q1;Q3)^b^	1.66 (0.97–2.85)	1.73 (0.96–2.08)	1.16 (1.15–1.16)	0.874*
Dialysis (yes/no; %)	62/34 (64.6/35.4)	14/6 (70/30)	3 (100)	0.619**
Primary diagnoses %				
Cardiovascular disease	5 (6.2)	2 (11.1)	1 (33.3)	^c^
Hematooncological disease	3 (3.7)	2 (11.1)		
Gastrointestinal disease	33 (40.7)	4 (22.2)		
Lung disease	19 (23.5)	5 (27.8)	1 (33.3)	
Urogenital cancer	6 (7.4)	1 (5.6)		
Intraabdominal pathology, other Cancer, other	15 (18.4)	4 (22.2)	1 (33.3)	
Infectious variables				
White blood cell count (10^9^ l^−1^; median; Q1; Q3)^b^	13.8 (9.3–20.6)	15.5 (8.9–19.1)	15.6 (12.2–15.6)	0.961*
Procalcitonin concentration (μg l^−1^; median; Q1; Q3)^b^	3.97 (1.2–15.7)	3.46 (0.31–9.93)	0.5 (0.23–0.5)	0.699*
C-reactive protein concentration (g l^−1^; median; Q1; Q3)^b^	17.7 (9.1–27.3)	15 (8–21.5)	18.4 (0.5–18.4)	0.603*
Disease severity				
SAPS II^d^ (mean ± SD)^a^	35 (27–44)	38 (30–50)	48 (41–48)	0.107*
Hospital stay (d; mean ± SD)^a^	23 (12–35)	29 (11–56)	21 (9–21)	0.071*
30-day mortality (%)	33 (34.4)	8 (38.1)	2 (66.7)	0.103**

Biometric data, primary diagnoses, infectious variables, and disease severity of 120 septic patients. Data were documented at the time of first diagnosing sepsis

*Numbers; *p*-value based on One-Way-ANOVA;

**Numbers; *p*-value based on Pearson-Chi-quadrat tests

^a^mean ± standard deviation

^b^median with 25 and 75 % quartiles (median; Q1; Q3);

^c^no statistical test has been applied here

^d^Simplified Acute Physiology Score II

**Table 3:** Clinicopathologic characteristics of septic patients with genetic variants in the prolyl-hydroxlase 2 gene (SNP rs480902)

	*PHD2* CC	*PHD2* CT	*PHD2* TT	*p*-value
	*n* = 72	*n* = 44	*n* = 12	
Patients characteristics				
Gender (women/men; %)	23/49 (31.9/68.1)	14/30 (31.8/68.2)	7/5 (58.3/41.7)	0.185**
age (years; median; Q1; Q3)^b^	58 (45–70)	54.5 (46–65)	67 (55–78)	0.111*
height (cm; median; Q1; Q3)^b^	174 (165–180)	178 (169–182)	165 (160–175)	0.122*
body weight (kg; median; Q1; Q3)^b^	80 (70–90)	85 (65–96)	80 (70–86)	0.455*
BMI (kg/m^2^; median; Q1; Q3)^b^	26.2 (22.9–30.5)	27.8 (22.8–33)	27.8 (24–30.8)	0.759*
Heart rate (min^−1^; median, Q1; Q3)^b^	101 (92–124)	110 (92–135)	96 (72–125)	0.753*
Mean arterial blood pressure (mmHg; median; Q1; Q3)^b^	76 (63–88)	73 (63–87)	70 (54–87)	0.484*
creatinin serum concentration (mg dl^−1^; (median; Q1;Q3)^b^	1.45 (1.0–3.0)	1.48 (1.0–2.1)	1.95 (1.0–2.4)	0.506*
Dialysis (yes/no; %)	48/23 (67.6/32.4)	30/14 (68.2/31.8)	6/6 (50/50)	0.468**
Primary diagnoses %				
Cardiovascular disease	4 (6.5)	5 (13.2)	1 (10)	^c^
Hematooncological disease	4 (6.5)	2 (5.3)	1 (10)	
Gastrointestinal disease	22 (35.5)	10 (26.3)	5 (50)	
Lung disease	13 (20.9)	12 (31.5)	1 (10)	
Urogenital cancer	6 (9.7)	2 (5.3)	0	
Intraabdominal pathology, other Cancer, other	13 (20.9)	7 (18.4)	5 (20)	
Infectious variables				
White blood cell count (10^9^ l^−1^; median; Q1; Q3)^b^	15.0 (9.7–20.9)	12.1 (8.1–18.3)	12.3 (8.9–21.2)	0.632*
Procalcitonin concentration (μg l^−1^; median; Q1; Q3)^b^	3.6 (1.0–22.2)	3.5 (1.0–11.5)	14.9 (0.7–18.7)	0.789*
C-reactive protein concentration (g l^−1^; median; Q1; Q3)^b^	17.2 (8.6–23.9)	15.2 (9.7–24.7)	22.9 (7.2–39.4)	0.155*
Disease severity				
SAPS II^d^ (mean ± SD)^a^	37 (29–45)	37 (27–49)	37 (25–51)	0.941*
Hospital stay (d; mean ± SD)^a^	26 (10–44)	17 (12–31)	23 (12–33)	0.121*
30-day mortality (%)	27 (37.5)	18 (40.9)	4 (33.3)	0.876**

Biometric data, primary diagnoses, infectious variables, and disease severity of 128 septic patients. Data were documented at the time of first diagnosing sepsis

*Numbers; *p*-value based on One-Way-ANOVA;

**Numbers; *p*-value based on Pearson-Chi-quadrat tests

^a^mean ± standard deviation

^b^median with 25 and 75 % quartiles (median; Q1; Q3);

^c^no statistical test has been applied here

^d^Simplified Acute Physiology Score II

### Genotyping

*HIF-1α* SNP rs11549465 was genotyped using a commercially available Taqman assay (No.: 4351379; Applied Biosystems; Carlsbad, USA) for the context sequence GTTACGTTCCTTCGATCAGTTGTCA**[C/T]**CATTAGAAAGCAGTTCCGCAAGCCC. Vector NTI Advance 11 computer program (Invitrogen, Carlsbad, CA) was used to develop primers for genotyping of *PHD2* SNPs. The *PHD2* (T/C) SNP (rs480902) was resolved using PCR sense primer 5′-TTTTAAAGGAGTGGGGTA-3′ and anti-sense primer 5′-GGTTGTTGGAAATGTAGAAT-3′, while the *PHD2* (C/T) SNP (rs516651) was resolved using PCR sense primer 5′-GCAGTTTGACCTGCCAGTTTTGCT-3′ and anti-sense primer 5′-TCTGAAAGGGGTCCAGCAGC-3′. Genotyping was run on a qPCR (Real-Time PCR) System (StepOnePlus™, Applied Biosystem, Carlsbad, USA) at standard conditions (60 °C for 30 min followed by, 95 °C for 10 s, and 15 s at 92 °C and 50 cycles at 60 °C for 1 s).

### RNA preparation, qPCR

Whole blood HIF-1α mRNA-expression was analyzed by qPCR following RNA-extraction from the last 62 consecutive patients with severe sepsis included into the study [2, 6]. RNA-extraction was performed on patients’ blood samples using the RNeasy Midi Kit (Qiagen, Hilden, Germany) following manufacturers protocol. One microgram of total RNA was reverse-transcribed to cDNA (complementary deoxyribonucleic acid). As described previously, primers for qPCR were designed using NCBI primer blast (Rockville Pike, MD) and obtained from Invitrogen (Invitrogen AG; Karlsruhe, Germany) [2].

First, qualitative PCR was performed for GAPDH (glyceraldehyde-3-phosphate-dehydrogenase) as a housekeeping gene to test the quality of cDNA synthesis [2, 14]. Second, quantitative PCR was performed to estimate the amount of specific cDNA for HIF-1α [2]. Resulting cDNA bands were visualized using ethidium bromide-stained 1.5 % (*w/v*) agarose gels [7].

HIF-1α mRNA expression was quantified by qPCR using SYBR® green as fluorescent dye (Eurogentec, Verviers, Belgium) on a StepOnePlus PCR system (Applied Biosystems, Carlsbad, USA). The denaturation steps were performed at 95 °C for 10 min, followed by 40 cycles at 95 °C for 15 s, and at 60 or 61 °C for 1 min, as described previously [2]. Amounts of specific cDNA were finally normalized to GAPDH using the ΔΔct method, and results are depicted as 2^ΔΔct^ values, as described [2].

### Statistical analyses

Data are presented as median with quartiles unless indicated otherwise, and in case of categorical variables as numbers and percentages. Data are presented as means ± standard deviation (SD) unless indicated otherwise. The two tailed *t*-test for independent samples or, in case of violations of the normality assumption (as tested by Kolmogorov-Smirnov and Shapiro-Wilk tests), the Wilcoxon signed rank test was used. Potential deviations from the Hardy-Weinberg equilibrium were ruled out using Court Lab calculator (Court MH; Court-laboratory Hardy-Weinberg calculator; Tufts University; www.tufts.com) for MS Excel 2011 software (Microsoft Deutschland, Unterschleißheim, Germany).

Furthermore, we investigated associations between the patients’ clinicopathologic characteristics and of the *HIF-1α* and *PHD2* genotypes with 30-day survival, defined as the interval from time of diagnosis of severe sepsis until day 30 or death, whichever occurred first. Patients alive after the 30-day follow-up were regarded as censored. Kaplan-Meier plots were used to display the overall 30-day survival data in the respective subcohorts followed by log-rank tests for comparison of the different genotypes. Thereafter, we computed hazard ratios and 95 %-CI using multivariate Cox regression models to assess the impact of the respective genotypes (*HIF-1α* and *PHD2* rs516651/rs480902), sex, age, Simplified Acute Physiology Score II, and requirement for dialysis as predictors for the clinical outcome (30-day survival). The proportional hazards assumption was checked for all variables included in the models and no deviation was found (data not shown).

An a priori alpha error p of less than 0.05 was considered statistically significant. In case of more than two groups one-way ANOVA with post hoc Student’s *t*-test and correction for multiple testing (*p* = 0.05/n) was used. Interdependence between HIF-1α-mRNA-expression and age was analyzed using Pearson correlation and linear regression analysis.

Statistical analyses were performed using SPSS 21.0 (SPSS Inc., Chicago, IL) and Graph Pad Prism 5 (Graph Pad Software, La Jolla, CA) software. Cox regression models were analysed with SAS Version 9.4 PHREG procedure (SAS Institute, Cary, NC, USA).

## Results

In this prospective observational trial we included 128 Caucasian septic patients of which 49 (38.3 %) died within 30 days irrespective of genotype.

### Hypoxia inducible factor-1α (C/T rs11549465) polymorphism

**Fig. 1:**
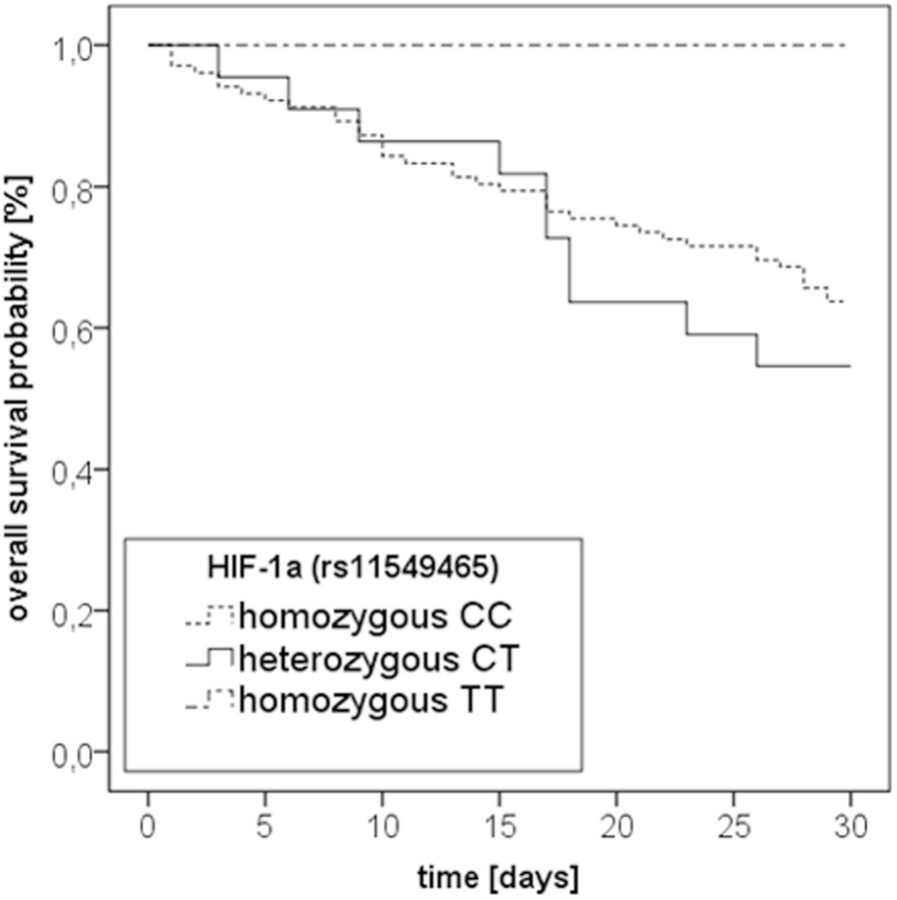
Kaplan-Meier plot for 30-day survival in 128 patients with severe sepsis stratified by HIF-1α (rs11549465) genotypes. Kaplan-Meier estimators for the three subgroups for all 128 patients. CC = homozygous CC genotype (*n* = 102); CT = heterozygous CT genotype (*n* = 22); TT = homozygous TT genotype (*n* = 1). For three patients, genotyping results were ambiguous, thus patients were excluded from analysis; Log-Rank-test: *p* = 0.591

Frequencies were 0.8 % for homozygous TT-genotypes, 17.6 % for heterozygous CT-genotypes, and 81.6 % for homozygous CC-genotypes (Table 1). 37 CC-genotypes (36.3 %) and 10 out of 22 CT- genotypes (45.5 %) died within 30 days of study inclusion (*p* = 0.502). For 3 patients, genotyping results were ambiguous, thus patients were excluded from analysis. Kaplan-Meier-graphs (Fig. 1) and multivariate Cox-regression-analyses (Table 4) showed no influence of this genetic variation on 30-day mortality from septic shock. Of note, only a single patient (survivor) carried the homozygous TT-genotype, as shown in Table 1 and Fig. 1. However, this single individual was not included into the statistical analyses.

**Table 4:** Cox regression analyses of septic patients with genetic variants in the Hypoxia-inducible factor-1α

	HR (95 %-CI)	*p*-value
rs11549465, CT vs. CC	1.42 (0.68–2.96)	0.350
rs11549465, TT vs. CC	NA^a^	NA^a^
Age, per year	1.023 (1.001–1.045)	0.040
Gender, male vs. female	1.70 (0.87–3.30)	0.120
Dialysis, yes vs. no	2.56 (1.15–5.67)	0.021
SAPS II, per point	1.02 (1.00–1.04)	0.059

Analysis set *N* = 121. Missing data: rs11549465 genotype (*n* = 3), dialysis (*n* = 1), SAPS II (*n* = 3)

^a^Only *n* = 1 in the TT group, this patient was censored, thus no HR could be estimated

**Fig. 2:**
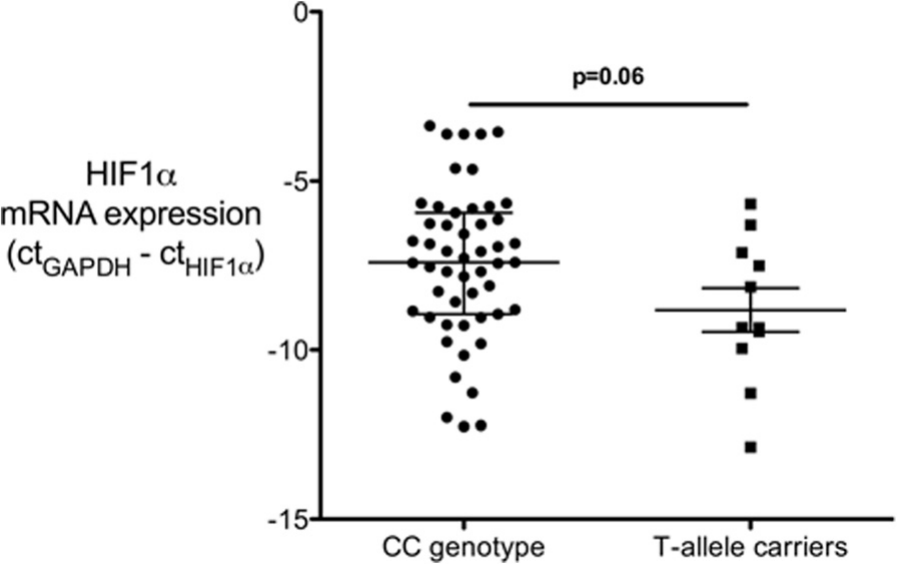
Hypoxia inducible factor 1α (HIF-1α) mRNA expression in 51 CC-genotypes and 11 T-allele-carriers. HIF-1α mRNA expression showed a trend towards lower values in T-allele-carriers (*n* = 11) compared to the homozygous CC-genotype (*n* = 51); *p* = 0.06

We then analyzed in 62 samples from consecutive septic patients whether this genetic variant influences HIF-1α-mRNA-expression. Of these patients 17.7 % (*n* = 11) were T-allele carriers of the HIF-1α SNP rs11549465, and 82.3 % were homozygous CC-genotypes (51 patients). HIF-1α-mRNA-expression was decreased by 62 % in T-allele carriers compared to CC-genotypes (Δct-values: T-allele carriers −8.82 ± 2.1 vs. CC-genotypes −7.45 ± 2.3, Fig. 2) but this was of borderline statistical significance *p* = 0.06), with CT-allele carriers being older (68 years; Q3; 53–72) than individuals with a CC (54 years; Q1-Q3; 48–66) genotype (*p* = 0.04). Of note, HIF-1α-mRNA-expression did not correlate with age (Pearson correlation: *r* = −0.1, *p* = 0.42), and HIF1-α-mRNA expression did not differ between genotypes even after adjusting for age (data not shown).

Further clinicopathogenic characteristics are displayed in Table 1 and did not differ between groups.

### Prolyl-hydroxlase 2 (C/T; rs516651) polymorphism

**Fig. 3:**
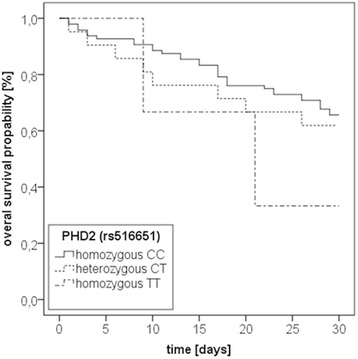
Kaplan-Meier plot of 30-day mortality of patients with severe sepsis stratified by prolyl-hydroxylase 2 (PHD 2; rs516651) genotypes. Kaplan-Meier estimators for the three subgroups for all 128 patients. CC = homozygous CC genotype (*n* = 96); CT = heterozygous CT genotype (*n* = 21); TT = homozygous TT genotype (*n* = 3); For eight patients genotyping results were ambiguous, thus patients were excluded from analyses; Log-Rank-test: *p* = 0.415

Frequencies were 2.5 % for the homozygous TT-genotype, 17.5 % for the heterozygous CT-genotype, and 80 % for the homozygous CC-genotype (Table 2). 33 CC-genotypes (34.4 %), 8 CT- genotypes (38.1 %) and 2 TT-genotypes (66.7 %) died within 30 days of study inclusion (*p* = 0.103). For 8 patients genotyping results were ambiguous, thus patients were excluded from further analyses. Kaplan-Meier-curve analysis (Fig. 3) and multivariate Cox-regression-analysis (Table 5) showed no influence of this genetic variation on 30-day mortality from septic shock. Patients clinicopathogenic characteristics did not differ between groups (Table 2).

**Table 5:** Cox regression analyses of septic patients with genetic variants in the prolyl-hydroxlase 2 gene (rs516651)

	HR (95 %-CI)	*p*-value
rs516651, CC vs. TT	1.15 (0.50–2.66)	0.730
rs516651, CT vs. TT	1.52 (0.35–6.65)	0.570
Age, per year	1.017 (0.99–1.040)	0.120
Gender, male vs. female	1.71 (0.87–3.36)	0.120
Dialysis, yes vs. no	1.99 (0.90–4.38)	0.087
SAPS II, per point	1.015 (0.99–1.04)	0.180

Analysis set: *N* = 116. Missing data: rs516651 genotype (*n* = 8), dialysis (*n* = 1), SAPS II (*n* = 3)

### Prolyl-hydroxlase 2 (T/C; rs480902) polymorphism

**Fig. 4:**
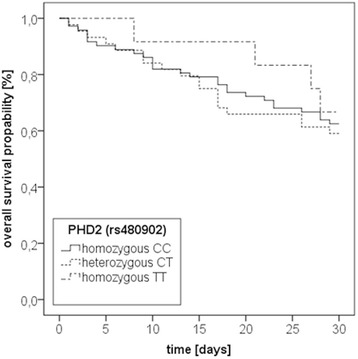
Kaplan-Meier plot of 30-day mortality of 128 patients with severe sepsis stratified by prolyl-hydroxylase 2 (PHD; rs480902) genotypes. Kaplan-Meier estimators for the three subcohorts. CC = homozygous CC genotype (*n* = 72); CT = heterozygous genotype (*n* = 44); TT = homozygous TT genotype (*n* = 12); *p* = 0.802

Frequencies were 9.4 % for the homozygous TT-genotype, 34.4 % for the heterozygous CT-genotype, and 56.3 % for homozygous CC-genotype (Table 3). 27 CC-genotypes (37.5 %), 18 CT- genotypes (40.9 %) and 4 TT-genotypes (33.3 %) died within 30 days of study inclusion (*p* = 0.876). Kaplan-Meier-curve analysis (Fig. 4) and multivariate Cox-regression-analysis (Table 6) showed no influence of this genetic variation on 30-day mortality from septic shock. However, the adjusted hazard ratio for mortality was 1.39 (95 %-CI 0.47–4.10) for CC vs. TT genotypes and 1.8 (95 %-CI 0.58–5.59) for CT vs. TT genotypes, suggesting a slight increase in 30-day mortality. Patients’ clinicopathogenic characteristics did not differ between groups (Table 3).

**Table 6:** Cox regression analyses of septic patients with genetic variants in the prolyl-hydroxlase 2 gene (rs480902)

	HR (95 %-CI)	*p*-value
rs480902, CT vs. TT	1.39 (0.47–4.10)	0.550
rs480902, CC vs. TT	1.80 (0.58–5.59)	0.310
Age, per year	1.025 (1.003–1.048)	0.025
Gender, male vs. female	1.62 (0.85–3.10)	0.140
Dialysis, yes vs. no	2.06 (0.98–4.30)	0.056
SAPS II, per point	1.018 (0.99–1.038)	0.074

Analysis set: *N* = 124. Missing data: dialysis (*n* = 1), SAPS II (*n* = 3)

## Discussion

In this prospective study we show, that allele frequencies in Caucasian for the functionally active SNPs of the hypoxic-inflammatory response, namely *HIF-1α* SNP rs11549465, *PHD2* rs516651 and rs480902 highly differ from Asian ethnicities. However these SNPs did not have a major impact on 30-day mortality in patients with severe sepsis in our sample. Of note, HIF-1α-mRNA-expression showed a trend towards lower expression in HIF-1α T-allele carriers.

*HIF-1α* SNP frequencies in our Caucasian cohort with sepsis revealed only a single homozygous TT-genotype carrier (0.8 %), whereas 17.6 and 81.6 % carried the heterozygous CT- and homozygous CC-genotypes, respectively. Notably, in people from India the TT-genotype is common with a frequency of 25 %, and 50 % CT-genotypes. T-allele carriers show an association with worse outcomes in metastatic prostate cancer or acute kidney injury [3, 15]. In this prospective study we could not confirm an influence of this SNP on 30-day mortality from severe sepsis. While this might relate to the very low frequency of homozygous TT-genotype carrier in our cohort, 30-day survival also did not differ between *HIF-1α* CT- and CC-genotypes.

However, HIF-1α mRNA expression showed a trend towards lower expression in T-allele carriers and on average was decreased by 62 %. As we reported previously, decreased HIF-1α mRNA expression inversely correlates with sepsis severity, i.e., with greater HIF-1α mRNA expression and intracellular HIF-1α protein suppression in patients with higher Simplified Acute Physiology Score II (SAPS II) [2]. As a high SAPS II is associated with increased mortality, one might expect increased mortality in *HIF-1α* T-allele carriers as well, however one might emphasize that sepsis itself contributes stronger to alterations in HIF-1α mRNA expression than this genetic variant does [13]. However, sepsis is a heterogeneous disease with multiple factors contributing to the patients’ mortality. Even, in our previous study HIF-1α mRNA-expression correlated with disease severity but not with outcome [2].

In a next step we analyzed genetic variants in the HIF-1 degrading *PHD2* gene, as these SNPs are associated with an increased oxygen saturation and heart rate in response to hypoxia and thus improved adaptation [5]. Furthermore, a PHD2 knockout in mice inhibits TNFα and ICAM-1 expression with reduced cell apoptosis and macrophage infiltration [11]. Furthermore, inhibition of PHD2 attenuates cardiac dysfunction in mice undergoing a high fat diet by a mechanism involving suppression of the MYD88/nuclear factor Kappa B inflammatory pathway [11]. Thus, functionally active genetic variants in the *PHD2* gene could also impact on patients outcome in severe sepsis, e.g., by altering the inflammatory response.

Therefore, we determined the genotype frequencies of *PHD2* SNP rs516651 carriers in our Caucasian cohort, with homozygous TT- genotypes found in 2.5 % of septic patients and heterozygous CT-genotypes in 17.5 %. Thus, we show that this SNP is present in Caucasians whereas in Tibetan high altitude residents, T-allele carriers do not exist [5]. Given this distribution in Caucasians, the *PHD2* SNP rs516651 turned out not to be an independent predictor for 30-day mortality in a multivariate Cox-regression analysis including common known risk factors for sepsis mortality in our rather small patients cohort.

The *PHD2* SNP rs480902, was common in Caucasians as well, however frequencies for homozygous TT-genotypes (9.4 %) and CT-genotypes (34.4 %), were lower when compared to Tibetan high altitude residents with frequencies of T-allele carriers of up to 75 % [5].

However, the *PHD2* SNP rs480902 genotype did not impact on 30-day mortality. PHD2 is a key-regulator of HIF-1α in erythropoietin producing cells and, therefore, crucial for regulation of erythropoietin secretion [16]. In mice, PHD2 deficiency leads to pronounced erythrocytosis due to a suppressed HIF-1α degradation and hence activation of the erythropoietin pathway, [16] as well as to increased angiogenesis [17]. Whereas regulation of erythrocytosis usually represents monocausal mid to long-term changes, severe sepsis is an acute life-threatening disorder depending on many issues. This might explain why *PHD2* genetic variants did not impact on survival in our cohort.

Our study has limitations: First, given the low genotype frequencies for the homozygous *HIF-1α* and *PHD2* rs516651 TT-genotype, 128 septic patients still need to be considered a small sample, reducing the explanatory power of our analysis. Thus, reanalyzing the influence of *HIF-1α* and *PHD2* SNPs in a larger cohort of septic patients is warranted. Furthermore, in this study we did not analyze the impact of either genetic variant on the patients phenotype i.e. regarding immune cell function or blood coagulation. This is of particular interest as other genetic variants, i.e. the NF*K*B1 promoter polymorphism -94ins/delATTG, turned out to be an independent predictor for 30-day mortality from septic shock, in a similar patients cohort of 143 patients [18]. In detail, the Deletion-allele was associated with altered blood coagulation and hyperinflammation [18]. Second, due to ethics issues and restrictions we only could analyze HIF-1α mRNA expression from the last 62 consecutive patients only at one time point. Analyzing HIF-1α mRNA expression in the course of sepsis depending on genetic variants might help to understand the regulation of the hypoxic-inflammatory pathway in the critically ill. More important, leukocyte HIF-1α mRNA expression was suppressed in T-allele carriers when compared to the CC-genotype, but this failed to reach statistical significance (*p* = 0.06). However, such variations in HIF-1α mRNA expression in T-allele carriers, are in accordance with the large variation of leukocyte HIF-1α expression in septic patients, [2] as well as a worse outcome of T-allele carriers with metastatic prostate cancer [6]. Third, septic patients are a very heterogeneous cohort due to various entities of sepsis or comorbidities. Thus, it might be favorable to analyze these polymorphisms in a cohort with a more homogenous sepsis entity, e.g., in patients with acute respiratory distress syndrome.

## Conclusions

In conclusion, genetic variants in *HIF-1α* and *PHD2* genes exist in Caucasians. However, homozygous TT-genotypes are infrequent and range from 0.8 % for *HIF-1α* SNP rs516651 to 9.4 % for *PHD2* SNP rs480902. Furthermore, even in our rather small sample, T-allele carriers of the *HIF-1α* SNP show a trend towards further suppressed HIF-1α mRNA expression compared to CC genotypes. However, neither of the analyzed genetic variants of *HIF-1α* and *PHD2* genes turned out to be independent risk factors for 30-day mortality of severe sepsis. Reanalyzing the influence of these genetic variants in a larger and more homogenous patients cohort i.e. patients with Acute respiratory distress syndrome (ARDS) is warranted to confirm the findings of our study.

## Abbreviations

HIF-1α, hypoxia-inducible-factor-1α; PHD, HIF-1 degrading prolyl-hydroxylases; SAPSII, simplified acute physiology score II; SNP, single nucleotide polymorphism
